# Clinical insights into the role of bepridil in recurrence prevention after ablation of persistent atrial fibrillation

**DOI:** 10.1002/joa3.70083

**Published:** 2025-05-05

**Authors:** Moyuru Hirata, Koichi Nagashima, Ryuta Watanabe, Yuji Wakamatsu, Naoto Otsuka, Shu Hirata, Masanaru Sawada, Yuji Saito, Sayaka Kurokawa, Kenta Murotani, Yasuo Okumura

**Affiliations:** ^1^ Division of Cardiology, Department of Medicine Nihon University School of Medicine Tokyo Japan; ^2^ Biostatistics Center Kurume University School of Medicine Kurume Fukuoka Japan

**Keywords:** atrial fibrillation, balloon ablation, catheter ablation, radiofrequency ablation

## Abstract

**Background:**

The role of bepridil in preventing atrial fibrillation (AF) recurrence following ablation for persistent atrial fibrillation (PerAF) remains uncertain, particularly in patients with severe atrial substrates.

**Methods:**

This retrospective study included 232 consecutive PerAF patients who underwent AF ablation between 2014 and 2019. Among them, 162 received bepridil for 3 months post‐ablation (Bepridil group), while 70 received no antiarrhythmic drugs (No‐AADs group). Baseline characteristics, procedural details, and outcomes were compared. Kaplan–Meier analysis and Cox regression models were used to evaluate AF/atrial tachycardia (AT) recurrence, with bepridil use treated as a time‐dependent covariable.

**Results:**

The Bepridil group had a higher body mass index (25.1 ± 3.7 vs. 23.8 ± 3.9), a higher prevalence of patients with a LAD >40 mm and a LAV >50 mL (67.9% vs. 47.1%, 64.2% vs. 48.5%, respectively), and lower left atrial appendage flow velocity (37.6 ± 15.0 vs. 42.7 ± 20.5 cm/min). They more frequently underwent intracardiac atrial cardioversion (61.7% vs. 40.0%) and additional extra‐pulmonary vein ablation (35.2% vs. 15.7%), but were less likely to receive balloon‐based ablation (39.5% vs. 62.9%) (*p* < 0.05 for all comparison). During a median follow‐up of 23.3 months, AF/AT‐free survival at 2 years was comparable between the Bepridil and No‐AADs groups (80.4% vs. 81.7%; *p* = 0.61). This finding remained consistent after adjusting for baseline characteristics and considering bepridil as a time‐dependent covariable. No bepridil‐related adverse events occurred.

**Conclusion:**

Bepridil may have a limited role in preventing AF/AT recurrence in PerAF patients, particularly those with severe atrial substrates. However, its overall impact appears to be small, warranting further investigation.

## Graphical abstract

This retrospective study included 232 consecutive persistent atrial fibrillation patients according to the inclusion criteria. Despite patients characteristics with more severe atrial substrates in the Bepridil group, Kaplan–Meier curves showed no significant difference in the AF/AT freedom rate and clinical adverse events between the Bepridil and No‐AADs groups. This finding remained consistent after adjusting for baseline characteristics and considering bepridil as a time‐dependent covariable. No adverse events related to bepridil were observed during the study.

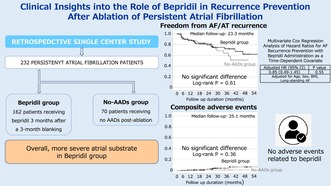

## 1 INTRODUCTION

Pulmonary vein isolation (PVI) is the cornerstone ablation strategy for paroxysmal atrial fibrillation (PAF), demonstrating superior efficacy in maintaining sinus rhythm compared with anti‐arrhythmic drug (AAD) therapy.^1^, ^2^ For patients with persistent atrial fibrillation (PerAF) and long‐standing PerAF, substrate modification techniques, such as complex fractionated atrial electrogram (CFAE) ablation, linear ablation, and non‐PV trigger ablation, have garnered clinical interest over the past decade as potential strategies to improve ablation outcomes.^3^, ^4^, ^5^

However, findings from the Substrate and Trigger Ablation for Reduction of Atrial Fibrillation Trial Part II (STAR‐AF2) revealed that substrate modification does not significantly reduce the recurrence of PerAF.^5^ Additionally, randomized controlled trials (RCTs) have failed to demonstrate the superiority of combining PVI with linear or CFAE ablation over PVI alone in PerAF patients.^6^, ^7^ Notably, one RCT with a 3‐year extended follow‐up suggested that linear ablation may reduce arrhythmia recurrence but is associated with a higher risk of iatrogenic atrial tachycardias (ATs).^8^ These findings underscore the challenges in achieving optimal outcomes in PerAF patients with advanced left atrial (LA) remodeling.

In this context, pharmacological therapy remains an area of interest. Continued use of previously ineffective antiarrhythmic drugs (AADs) has been shown to significantly reduce AT recurrence during the first year after PVI in PAF patients.^9^ Among AADs, bepridil stands out for its unique pharmacological profile. Originally developed for arrhythmias and later used for angina because of its calcium channel blocking effects, bepridil has been identified as highly effective in facilitating pharmacological defibrillation and maintaining sinus rhythm in patients with long‐standing PerAF.^10^ Importantly, bepridil is currently approved for use only in Japan, where it is prescribed for refractory atrial fibrillation (AF) and angina. In contrast, it has been withdrawn from the market in Western countries because of safety concerns, particularly its association with QT prolongation and torsades de pointes.

Given its pharmacological benefits and unique availability in Japan, this study aims to evaluate the potential role of bepridil in reducing AF/AT recurrence in PerAF patients following catheter ablation, using a retrospective cohort design.

## 2 METHODS

### 2.1 Study design

**FIGURE 1:**
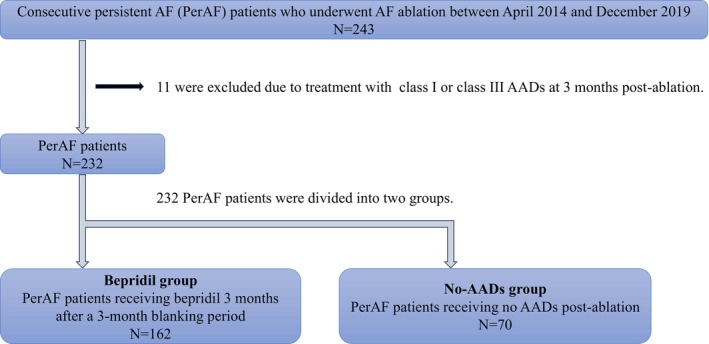
Inclusion criteria.

This retrospective observational study included 243 patients with PerAF who underwent initial AF ablation at Nihon University Itabashi Hospital between 2014 and 2019. At our institution, patients with PerAF generally did not receive class I or class III AADs, including amiodarone, postablation. Therefore, the inclusion criteria were patients treated with either bepridil or no AADs 3 months after ablation, while those treated with class I or class III AADs at 3 months were excluded. PerAF was defined as sustained AF episodes lasting ≥7 days, including long‐standing PerAF (>1 year duration). Of the 243 patients, 11 were excluded because of treatment with other AADs at 3 months postablation. The remaining 232 patients were divided into two groups: 162 patients receiving bepridil 3 months after a 3‐month blanking period (Bepridil group) and 70 patients receiving no AADs postablation (No‐AADs group) (Figure 1). The decision to continue or discontinue bepridil postablation was made at the discretion of the treating physician, based on clinical judgment, including patient symptoms and perceived arrhythmic risk.

### 2.2 Echocardiographic parameters

In all patients, transthoracic echocardiography was performed within 7 days before ablation. Left atrial diameter (LAD), left atrial volume (LAV), E/e', and left ventricular ejection fraction were measured. LAV was calculated using the ellipsoid formula based on three LAD measurements: LAV = /6 × LAD_1_ × LAD_2_ × LAD_3_, where LAD_1_ is the M‐mode measurement, and LAD_2_ and LAD_3_ are measurements of the short and long axes in the apical four‐chamber view. In all patients, transesophageal echocardiography was also performed prior to the procedure to exclude thrombus, and left atrial appendage (LAA) flow velocity was measured.

### 2.3 AF ablation protocol

All AADs were discontinued for at least five half‐lives. The procedure was performed with patients under deep sedation with dexmedetomidine and fentanyl. After a single transseptal puncture, two long sheaths (Agilis steerable sheath and SL0 sheath; Abbott, Inc., Chicago, IL) were inserted into the LA via a transseptal puncture. An activated clotting time >300 s was maintained by heparin during the procedure. The 3D geometry of the LA and PVs was created using the CARTO3 (Biosense Webster, Diamond Bar, CA) or Ensite NavX mapping system (Abbott). As for RF ablation, extensive encircling PVI was performed by an irrigated‐tip RF ablation catheter (3.5‐mm tip, Navistar ThermoCool SmartTouch, Biosense Webster; or TactiCath, Abbott) under the guidance of ablation index (AI) or lesion size index (LSI). Target FTI was 450 for the anterior sites and 350–400 for the posterior sites with the power setting of 30–45 W,^11^ target AI and LSI were 450 and 5.5 for the anterior sites and 400 and 4.5–5.0 for the posterior sites with the power of 40–45 W, respectively.^12^ Extra‐pulmonary vein (PV) LA ablation, including CFAE‐guided ablation in LA,^13^, ^14^ liner ablation, and/or non‐PV foci ablation, was performed in cases in which AF sustained even after PVI depending on the operator's discretion.

Regarding balloon‐based ablations, Ensite NavX mapping system was used to guide LA‐PV mapping. Cryoballoon ablation (CBA) was performed with a 28‐mm cryoballoon (ARC‐Adv‐CB, Arctic Front Advance; Medtronic, Inc., Minneapolis, MN) and cryothermal energy was applied to each PV for 180 s and then for 120 s. Hot balloon ablation (HBA) was performed with a hot balloon (SATAKE HotBalloon; Toray Industries, Inc., Tokyo, Japan) and radiofrequency‐generated thermal energy was applied to the right superior PV antrum for 210 s, left superior PV antrum for 240 s, and right and left inferior PV antra for 150 s. In both CBA and HBA, catheter ablation for atrial fibrillation using a balloon catheter is basically performed as PVI in our institution unless non‐PV triggers occur frequently. If AF was persisted after ablation procedure, intracardiac atrial cardioversion (IACV) was performed. Regardless of the use of RF ablation, CBA or HBA, cavotricuspid isthmus (CTI) ablation was performed in the cases in which common atrial flutter (AFL) was clinically documented or incidentally lasted for 1 min during procedure.

### 2.4 Follow‐up

Postablation AAD was determined at the operators' discretion, based on individual patient characteristics. In the Bepridil group, bepridil therapy was resumed after the ablation procedure and continued for at least 3 months. In the No‐AADs group, either no AADs were administered after ablation, or any AADs that were resumed were discontinued within 3 months postablation. This classification was based on the treatment status at 3 months following the blanking period. All patients underwent routine follow‐up at our institution at 3 weeks, and at 3, 6, 12, and every 6 or 12 months postablation, or whenever they experienced any symptoms. During each visit, 12‐lead electrocardiograms were recorded, and 24‐h Holter monitoring was performed at 3, 6, 12, and every 12 months postablation. Recurrence was defined as any documented AF or AT lasting more than 30 s during the follow‐up period.

### 2.5 Study outcomes

The primary effectiveness outcome was the AF/AT freedom rate throughout the entire follow‐up period after a 3‐month blanking period. The primary safety outcome was procedure‐related complications occurring within 2 days of the ablation. The secondary outcome was the occurrence of composite adverse events including stroke, heart failure, acute coronary syndrome, sick sinus syndrome, major bleeding, and all‐cause death during the entire follow‐up period after ablation.

### 2.6 Statistical analysis

Continuous variables were expressed as mean ± standard deviation (SD) values or as median and interquartile range (IQR). Dichotomous variables were presented as the number and percentage of cases. Differences in the continuous variables were analyzed by the Student's *t*‐test or Mann–Whitney *U* test, as appropriate. The Chi‐square test was used to analyze the differences in the dichotomous variables unless the expected values in cells were <5, in which case a Fisher's exact test was used. AF/AT recurrence‐free survival and composite adverse event curves were generated by the Kaplan–Meier method and compared using a log‐rank test between the Bepridil group and the No‐AADs group. Univariate Cox regression analysis for AF/AT recurrence was conducted. Variables potentially related to postablation AF/AT recurrence, including age, gender, body mass index (BMI), AF duration (<1 year or ≥1 year), LAV, extra‐PV LA ablation, and IACV required postablation, were subsequently included in a multivariate Cox regression analysis. Additionally, prespecified subgroup analyses stratified by patient characteristics were performed. In all three analyses, bepridil administration was treated as a time‐dependent covariate. All statistical analyses were conducted using JMP Pro 16 software (SAS Institute, Cary, NC), SAS 9.4 software (SAS Institute, Cary, NC), or SPSS Statistics 29 (IBM Corp., Armonk, NY, USA) software. A *p*‐value of <0.05 was considered statistically significant.

## 3 RESULTS

### 3.1 Patient and ablation procedure characteristics

The characteristics of the patients and the ablation procedures are detailed in Table 1. The BMI was significantly higher in the Bepridil group (25.1 ± 3.7 vs. 23.8 ± 3.9 kg/m^2^; *p* = 0.018). The prevalence of patients with a LAD >40 mm and a LAV >50 mL was significantly higher in the Bepridil group than in the No‐AADs group (LAD >40 mm, 110 [67.9%] vs. 33 [47.1%], *p* = 0.002; LAV > 50 mm, 104 [64.2%] vs. 34 [48.5%], *p* = 0.026). The Bepridil group demonstrated a significantly lower LAA flow velocity compared with the No‐AADs group (37.6 ± 15.0 vs. 42.7 ± 20.5 cm/min, *p* = 0.041). Pre‐AADs were more frequently used in the Bepridil group (45.7% vs. 30.0%; *p* = 0.026); in particular, bepridil was mainly used (40.1% vs. 18.6%; *p* = 0.001). There were no significant differences in other characteristics of the patients between the two groups. Balloon ablation was more frequently performed in the No‐AADs group (39.5% vs. 62.9%; *p* ‐= 0.001). Extra‐PV LA ablation was more frequently performed (35.2% vs. 15.7%; *p* = 0.003) but IACV was more frequently required after ablation in the Bepridil group (61.7% vs. 40.0%; *p* = 0.002).

**TABLE 1:** Patient characteristics, echocardiographic parameters, medications, and procedural details in the Bepridil and No‐AADs Groups.

	Bepridil group (*n* = 162)	No‐AADs group (*n* = 70)	*p*‐value
Age (years)	62.7 ± 10.0	63.5 ± 11.7	0.59
Male gender	127 (78.4%)	54 (77.1%)	0.83
Height (cm)	167.2 ± 8.4	166.2 ± 9.8	0.42
Weight (kg)	70.5 ± 13.1	66.0 ± 13.3	0.020
BMI (kg/m^2^)	25.1 ± 3.7	23.8 ± 3.9	0.018
AF duration (months)	9 (5, 36)	11 (5, 41)	0.43
Long‐lasting PerAF (AF duration≥1 year)	69 (42.6%)	32 (45.7%)	0.81
Medical history			
HT	96 (59.3%)	38 (54.3%)	0.48
DM	26 (16.1%)	12 (17.1%)	0.84
HF	20 (12.4%)	15 (21.4%)	0.08
Vascular disease	9 (8%)	3 (4%)	0.27
Stroke/TIA	16 (9.9%)	6 (8.6%)	0.76
CHADS_2_ score	1 (0, 2)	1 (0, 2)	0.45
CHA_2_DS_2_‐VASc score	2 (1, 3)	2 (1, 3)	0.72
Echocardiographic variables			
LVEF (%)	63.7 ± 8.1	62.4 ± 11.4	0.31
LAD (mm)	42.4 ± 6.3	41.4 ± 7.0	0.32
LAD >40 mm	110 (67.9%)	33 (47.1%)	0.002
LAV (mL)	57.1 ± 18.6	53.4 ± 23.3	0.20
LAV >50 mL	104 (64.2%)	34 (48.5%)	0.026
E/e'	10.7 ± 4.2	10.4 ± 4.4	0.61
LAA flow (cm/min)	37.6 ± 15.0	42.7 ± 20.5	0.041
Pre‐ablation medications			
β‐blocker	61 (37.7%)	27 (38.6%)	0.89
Pre‐ablation AADs	74 (45.7%)	21 (30.0%)	0.026
Class I	9 (5.6%)	5 (7.1%)	0.93
Class III	3 (1.9%)	3 (4.3%)	0.28
Bepridil	65 (40.1%)	13 (18.6%)	0.001
Procedural characteristics			
Ablation modality			
RFCA	98 (60.5%)	26 (37.1%)	0.001
Balloon ablation	64 (39.5%)	44 (62.9%)	0.001
Extra‐PV LA ablation	57 (35.2%)	11 (15.7%)	0.003
CTI ablation	46 (28.4%)	29 (41.4%)	0.05
IACV required post‐ablation	100 (61.7%)	28 (40.0%)	0.002

*Note*: Values are expressed as mean ± SD, median (interquartile range), or number (%).

Abbreviations: AADs, antiarrhythmic drugs; AF, atrial fibrillation; BMI, body mass index; CTI, cavotricuspid isthmus; DM, diabetes mellitus; HF, heart failure; HT, hypertension; IACV, intracardiac atrial cardioversion; LAA, left atrial appendage; LAD, left atrial diameter; LAV, left atrial volume; LVEF, left ventricular ejection fraction; PV, pulmonary vein; RFCA, radiofrequency catheter ablation; TIA, transient ischemic attack.

### 3.2 Clinical outcomes and procedure‐related complications

**FIGURE 2:**
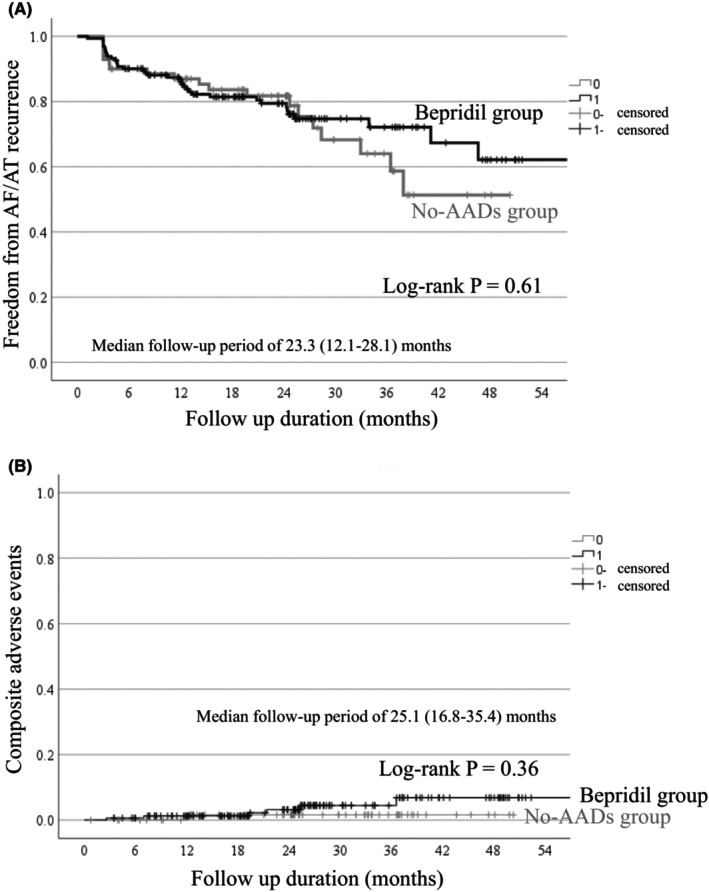
Kaplan–Meier curves for freedom from AF/AT recurrence (A) and composite adverse events (B).

**FIGURE 3:**
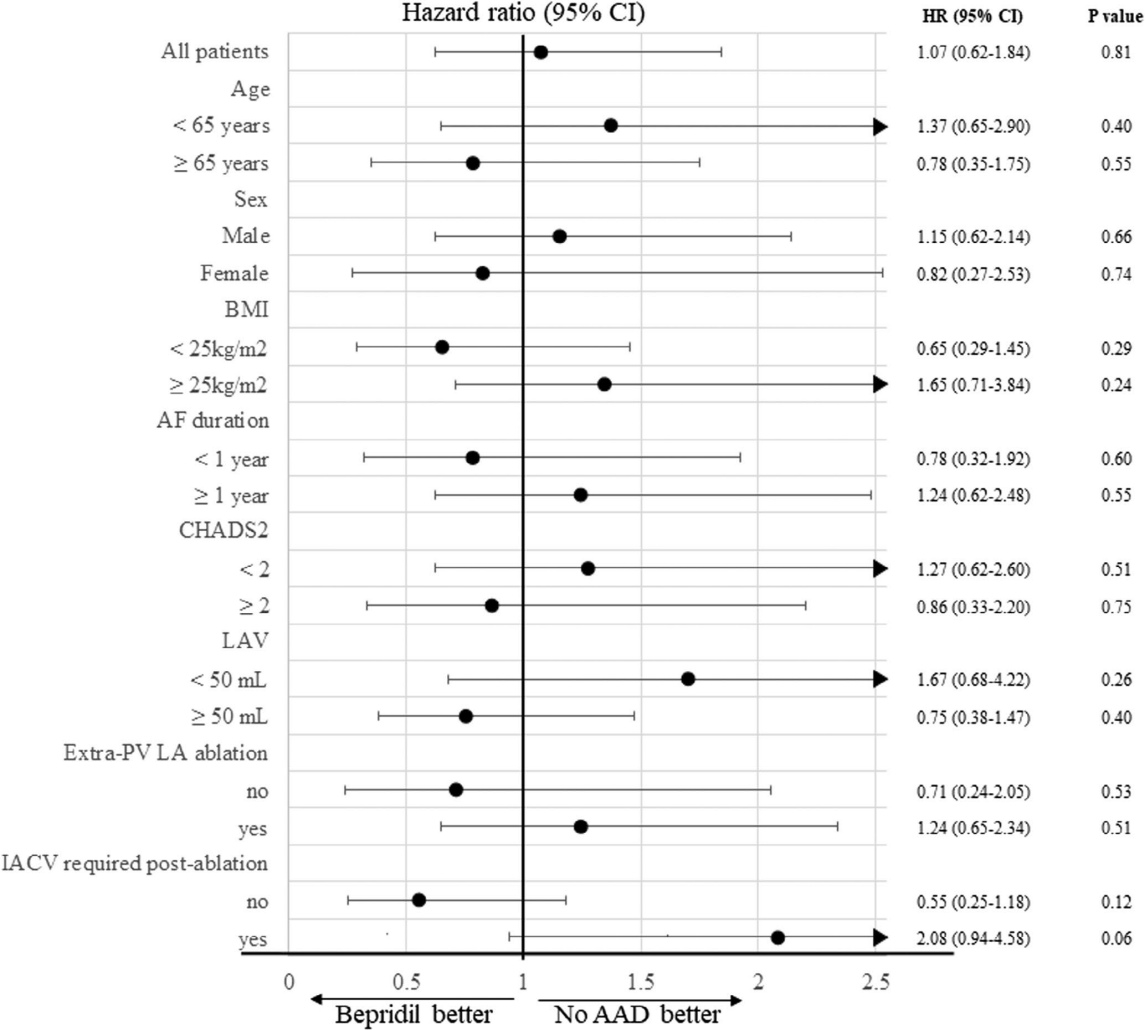
Hazard ratios and 95% confidence intervals for AF/AT recurrence prevention with bepridil administration in prespecified subgroups.

The long‐term AF/AT freedom rate is illustrated in Figure 2A. Despite differing patient characteristics, the AF/AT freedom rate did not differ between the Bepridil group and the No‐AADs group (80.4% vs. 81.7% at 2 years; *p* = 0.61) (Figure 2A). The complications associated with AF ablation and clinical adverse events during follow‐up are detailed in Table 2. There were no significant differences in the complications of AF ablation and clinical adverse events between the two groups. Univariate analysis identified long‐standing PerAF (AF duration >1 year) and LAV as significant clinical variables associated with AF/AT recurrence, whereas other variables, including bepridil administration, were not significant (Table 3). In a multivariate Cox regression model where bepridil administration was included as a time‐dependent covariate, it remained a nonsignificant factor for AF/AT recurrence (Table 4). Sub‐analysis stratified by patient characteristics revealed no significant beneficial effect of bepridil administration across any of the various subgroups. However, patients with BMI <25 kg/m^2^ or those not requiring IACV post‐ablation showed numerically lower hazard ratios (HR 0.65 [0.29–1.45], *p* = 0.29 and HR 0.55 [0.25–1.18], *p* = 0.12) (Figure 3).

**TABLE 2:** Procedural complications of catheter ablation for atrial fibrillation and clinical adverse events during follow‐up.

	Bepridil group (*n* = 162)	No‐AADs group (*n* = 70)	*p*‐value
Procedural complications of catheter ablation for atrial fibrillation			
Cardiac tamponade	2 (1.2%)	1 (1.4%)	0.66
Stroke	0	0	1.00
Sinus node dysfunction	1 (0.6%)	2 (2.9%)	0.23
Phrenic nerve palsy	1 (0.6%)	0	1.00
Gastrointestinal complication	1 (0.6%)	1 (1.4%)	0.51
Arteriovenous fistula	0	0	1.00
Pseudoaneurysm	1 (0.6%)	0	1.00
Clinical adverse events during follow‐up			
Stroke/TIA	0	0	1.00
Noncardiac death	1 (0.62%)	0	1.00
Heart failure	1 (0.62%)	0	1.00
Major bleeding	1 (0.62%)	0	1.00
Acute coronary syndrome	0	0	1.00
Sick sinus syndrome	0	1 (1.4%)	0.31

*Note*: Values are expressed as number (%).

Abbreviations: AADs, antiarrhythmic drugs; TIA, transient ischemic attack.

**TABLE 3:** Patient characteristics, medications, procedural details, and hazard ratios for AF/AT recurrence.

	AF/AT recurrence (*n* = 56)	No AF/AT recurrence (*n* = 176)	*p* value	HR (95% CI)	*p*‐value
Age (years)	62.7 ± 10.0	63.5 ± 11.7	0.82	1.00 (0.97–1.02)	0.77
Male sex	43 (76.8%)	138 (78.4%)	0.83	0.96 (0.52–1.79)	0.91
Height (cm)	167.3 ± 9.2	166.8 ± 8.8	0.72	1.01 (0.98–1.04)	0.57
Weight (kg)	70.5 ± 13.1	68.7 ± 13.4	0.36	1001 (0.99–1.03)	0.45
BMI (kg/m^2^)	25.1 ± 3.5	24.6 ± 3.9	0.37	1.02 (0.95–1.09)	0.62
AF duration (months)^a^	31 (6, 59)	9 (5, 30)	0.006	2.09 (1.32–3.31)	0.002
Long‐lasting PerAF	35 (62.5%)	66 (37.5%)	0.001	2.46 (1.43–4.24)	0.001
Medical history					
HT	32 (57.1%)	102 (58.0%)	0.91	0.94 (0.55–1.60)	0.82
DM	9 (16.1%)	29 (16.5%)	0.84	0.90 (0.44–1.83)	0.76
HF	10 (17.9%)	25 (14.2%)	0.51	1.50 (0.76–2.98)	0.25
Vascular disease	4 (7.1%)	10 (5.7%)	0.69	1.08 (0.39–2.98)	0.89
Stroke/TIA	4 (7.1%)	18 (10.2%)	0.49	0.94 (0.56–1.56)	0.80
CHADS_2_ score	1 (0, 2)	1 (0, 2)	0.91	1.01 (0.79–1.30)	0.92
CHA_2_DS_2_‐VASc score	2 (1, 3)	2 (1, 3)	0	0.97 (0.81–1.17)	0.74
Echocardiographic variables					
LVEF (%)	64.0 ± 7.7	63.1 ± 9.7	0.53	1.00 (0.98–1.03)	0.78
LAD (mm)	42.5 ± 6.6	41.9 ± 6.5	0.53	1.02 (0.98–1.07)	0.33
LAV (mL)	60.0 ± 23.0	54.9 ± 19.4	0.10	1.02 (1.00–1.03)	0.009
E/e'	11.1 ± 4.6	10.4 ± 4.1	0.30	1.04 (0.98–1.11)	0.19
Pre‐ablation medications					
β blocker	27 (48.2%)	61 (34.7%)	0.07	1.91 (1.12–3.24)	0.002
Pre‐ablation AADs	74 (45.7%)	21 (30.0%)	0.026	1.42 (0.84–2.40)	0.19
Class I	2 (3.4%)	12 (6.8%)	0.53	0.56 (0.14–2.30)	0.42
Class III	3 (5.4%)	3 (1.7%)	0.15	1.74 (0.54–5.58)	0.35
Bepridil	24 (42.9%)	54 (30.7%)	0.09	1.39 (0.82–2.36)	0.23
Post‐bepridil administration^b^	37 (66.1%)	125 (71.0%)	0.48	1.07 (0.62–1.84)	0.81
Procedural characteristics					
Ablation modality					
RFCA	30 (53.6%)	94 (53.4%)	0.98	1.15 (0.68–1.94)	0.61
Balloon ablation	26 (46.4%)	82 (46.6%)	0.98	1.07 (0.82–1.39)	0.61
Extra‐PV LA ablation	16 (28.9%)	52 (29.6%)	0.89	1.08 (0.60–1.92)	0.80
CTI ablation	20 (35.7%)	55 (31.3%)	0.54	1.00 (0.76–1.32)	0.99
IACV required post‐ablation	28 (50.0%)	76 (43.2%)	0.37	1.08 (0.64–1.83)	0.77

*Note*: Values are expressed as mean ± SD, median (interquartile range), or number (%).

Abbreviations: CI, confidence interval; long‐standing AF; AF duration ≥1 year; HR, hazard ratio. Other abbreviations are the same as in Table 1.

^a^ Log‐transformed AF duration was used to calculate HR.

^b^ Post‐bepridil administration was included as a time‐dependent covariate for HR analysis.

**TABLE 4:** Multivariate Cox regression analysis of hazard ratios for AF recurrence prevention with bepridil administration.

	HR (95% CI)	*p*‐value
Model 1 (age, gender, BMI, long‐standing AF)	0.85 (0.49–1.45)	0.55
Model 2 (age, gender, BMI, LAV)	0.90 (0.52–1.55)	0.70
Model 3 (age, gender, BMI, extra‐PV LA ablation)	0.84 (0.48–1.46)	0.54
Model 4 (age, gender, BMI, IACV required post‐ablation)	0.78 (0.45–1.36)	0.39

*Note*: Hazard ratios are presented for Bepridil administration versus the No‐AADs group.

Abbreviations: HR, hazard ratio; CI, confidence interval. Other abbreviations are the same as in Table 1.

### 3.3 QT prolongation and adverse events

The mean dose of the bepridil administered as a postablation AAD was 101 ± 32 mg, with a mean duration of 21 ± 4 months. Table 5 presented the QT and QTc intervals measured pre‐ablation and 12 months postablation. The pre‐ablation QT and QTc intervals were comparable between the two groups. However, at 12 months postablation, the QT and QTc intervals tended to be longer in the Bepridil group compared with the No‐AADs group (QT: 410 ± 41 ms vs. 400 ± 42 ms; *p* = 0.12, QTc: 447 ± 30 ms vs. 438 ± 32 ms; *p* = 0.049). There were no differences in composite adverse events between the Bepridil group and the No‐AADs group throughout the entire follow‐up period (Figure 2B).

**TABLE 5:** QT and QTc intervals measured pre‐ablation and 12 months post‐ablation.

	Bepridil group	No‐AADs group	*p*‐value
QT interval at pre‐ablation (ms)	376 ± 43	385 ± 45	0.15
QTc interval at pre‐ablation (ms)	439 ± 30	434 ± 31	0.30
QT interval at 12 months post‐ablation (ms)	410 ± 41	400 ± 42	0.12
QTc interval at 12 months post‐ablation (ms)	447 ± 30	438 ± 32	0.049

*Note*: Values are expressed as mean ± SD. Abbreviations are the same as in Table 1.

## 4 DISCUSSION

### 4.1 Main findings

This study provides 3 major insights: (1) Compared with the No‐AADs group, the prevalence of patients with a LAD >40 mm and a LAV >50 mL was higher, the Bepridil group demonstrated a lower LAA flow velocity, and required more IACV for AF termination, necessitating more extra‐PV LA ablation, which suggests a more severe atrial substrate, (2) Despite these patient characteristics, there were no significant differences in the long‐term AF/AT freedom rate between patients treated with bepridil and those not administered AADs after catheter ablation for PerAF. Bepridil's effect on outcomes remained nonsignificant after multivariate adjustment and subgroup analysis, and (3) Bepridil prolonged the QT interval, but no prolongation of Tpd was observed. Furthermore, there were no differences in long‐term composite adverse events between the two groups.

### 4.2 Long‐term effectivity and safety of bepridil after catheter ablation for persistent AF

In our study, patients in the bepridil group exhibited several indicators of more advanced atrial remodeling compared with those who did not receive AADs. Specifically, the proportion of patients with LAD >40 mm and LAV >50 mL was significantly higher in the bepridil group. In addition, these patients demonstrated a significantly lower LAA flow velocity and a higher requirement for IACV following ablation, despite undergoing more extensive LA ablation. These findings suggest that the bepridil group had more severe structural and functional remodeling of the LA substrate. Interestingly, despite these indicators of advanced remodeling and a higher baseline risk of recurrence, the long‐term freedom from AF/AT recurrence did not differ significantly between the bepridil and No‐AADs groups.

Two potential mechanisms may explain this observation. First, the more extensive LA ablation performed in the bepridil group may have helped counteract the effects of substrate remodeling. However, studies have shown that patients with PerAF who experience long‐term recurrence after catheter ablation often exhibit persistent atrial remodeling and the emergence of non‐PV triggers, even after extensive ablation.^15^ Insufficient substrate modification or inadequate PV isolation durability may also contribute to AF recurrence or iatrogenic AT occurrence.^8^, ^16^ These factors may diminish the effects of extra‐PV LA ablation, particularly over a prolonged follow‐up period. Indeed, the limited effectiveness of extra‐PV LA ablation has been demonstrated in studies such as the STAR‐AF2 trial, as well as in cases targeting areas with complex fractionated electrograms, as shown in the EARNEST‐PVI trial.^5^, ^6^, ^8^

Second, the pharmacologic properties of bepridil may have contributed to rhythm maintenance in a subset of patients. Bepridil inhibits the downregulation of L‐type calcium channels in atrial muscle cells, preventing ERP shortening and suppressing the progression of electrical remodeling in vivo.^17^, ^18^ However, when bepridil administration was evaluated as a time‐dependent covariate in a multivariate Cox regression model, it was not associated with a significant reduction in AF/AT recurrence. Subgroup analyses also did not identify any patient population in which bepridil showed a statistically significant benefit. Nonetheless, numerically lower hazard ratios were observed in patients with BMI <25 kg/m^2^ or those not requiring IACV, suggesting that bepridil might provide modest benefit in patients with less extensive remodeling. However, these trends did not reach statistical significance. Taken together, these findings suggest that the overall effectiveness of bepridil in preventing AF/AT recurrence following catheter ablation is limited. This may be owing to the overriding influence of advanced atrial remodeling or insufficient substrate modification, which may diminish the drug's impact. It is also possible that the dosage of bepridil used postablation was insufficient to achieve optimal reverse remodeling effects, as previous studies have indicated that its antiarrhythmic efficacy may be dose‐dependent.^10^, ^19^

Although a previous study by Kondo et al. suggested that postablation bepridil may reduce AF recurrence more effectively than other AADs, their analysis did not adjust for the duration of AAD administration.^20^ In contrast, our study accounted for the time‐dependent nature of bepridil exposure and conducted multivariate analyses accordingly. Additionally, the higher success rate observed in our cohort may reflect procedural advances, as their study was conducted several years earlier. These methodological and temporal differences may help explain the discrepancy in outcomes. Furthermore, Yakabe et al. reported that patients with PerAF who achieved pharmacological cardioversion with bepridil prior to ablation had relatively preserved atrial voltage and lower recurrence rates.^21^ However, our study did not mandate bepridil use before ablation, and electroanatomical mapping data (e.g., low voltage zone [LVZ] or LVZ ratio) were not available. This limits our ability to assess correlations between bepridil responsiveness and substrate quality. While Yakabe's concept is meaningful, our focus was on postablation bepridil use and its long‐term efficacy in routine clinical practice.

### 4.3 Adverse events including QT prolongation

It has been reported that patients who experience a recurrence of AF after catheter ablation are at a higher risk of cardiovascular events, including stroke and heart failure, compared with those who remain free of recurrence.^22^, ^23^ Therefore, patients with progressed LA remodeling may benefit from continuing bepridil postcatheter ablation to prevent AF recurrence. In this study, we did not observe any significant differences in adverse events between the bepridil group and the No‐AADs group, suggesting that its prognostic impact is, unfortunately, limited. Bepridil is a multi‐channel blocker that affects various ion channels, including rapid, slow, and ultrarapid delayed rectifier potassium currents, sodium currents, and L‐ and T‐type calcium currents. This pharmacological profile can lead to QT and QTc interval prolongation, increasing the risk of torsade de pointes (TdP).^24^ As a result, regular monitoring of the QT and QTc intervals is essential to prevent TdP. Notably, while QT interval prolongation was observed in this study, no TdP or other adverse events related to bepridil occurred during the follow‐up period. These findings suggest that a low dose of bepridil (101 ± 32 mg) is clinically safe and applicable.

### 4.4 Study limitations

There were several limitations to this study. It was a retrospective observational study with a relatively small sample size, conducted at a single center, which may limit its generalizability. Although univariate and multivariate models were used to adjust for confounding factors, residual confounding may persist because of the complexity of AF predictors and treatment heterogeneity. Treatment assignment—including bepridil administration and ablation technique—was made at the physician's discretion. This may have introduced selection bias. However, we addressed this through statistical adjustment using time‐dependent covariate modeling and by limiting the number of covariates to avoid overfitting, given the sample size and number of events. Moreover, univariate analysis demonstrated that ablation modality was not significantly associated with AF/AT recurrence, which supports the internal validity of our comparative analysis despite procedural differences between groups. Our study could not evaluate electroanatomical atrial substrate (e.g., LVZ or LVZ ratio) because of the lack of systematic voltage mapping. In contrast to the study by Yakabe et al., which showed that pre‐ablation bepridil responsiveness correlated with preserved atrial voltage, our protocol did not include uniform bepridil administration before ablation or evaluate pharmacological conversion.^21^ The follow‐up period had a median duration of 2 years, which provides valuable insights into long‐term outcomes but may still be insufficient to fully capture the extended effects or risks of bepridil. Additionally, the detection of AF recurrence may have been underestimated because of the reliance on periodic noninvasive monitoring rather than continuous monitoring. Moreover, variability in ablation strategies (radiofrequency vs. balloon ablation), bepridil dosage, and treatment duration could have influenced the results. Our study did not include newer ablation technologies, such as pulsed field ablation, which may yield different outcomes. Lastly, ongoing advancements in ablation techniques during the study period may limit the applicability of our findings to current clinical practice. Despite these limitations, our study provides a comprehensive evaluation of bepridil in a modern PerAF cohort using robust statistical methods, including time‐dependent covariate analysis. Further prospective studies are warranted to confirm our findings and clarify the role of bepridil in patients with advanced atrial remodeling.

## 5 CONCLUSIONS

Patients receiving bepridil after ablation required more IACV for AF/AT termination, even after LA substrate ablation, indicating more advanced atrial remodeling. Despite this, AF/AT recurrence rates were similar between the bepridil and No‐AADs groups. After adjusting for differences in patient characteristics, recurrence rates remained comparable, suggesting that its overall impact in this setting may be limited.

## CONFLICT OF INTEREST STATEMENT

No disclosures. The work was supported by departmental resources only.

## ETHICS STATEMENT

The studies involving human participants were performed according to protocols approved by the Institutional Review Board of Nihon University Itabashi Hospital (RK‐230214‐9).

## Data Availability

No data are available. Not applicable.

## References

[1] Morillo CA , Verma A , Connolly SJ , Kuck KH , Nair GM , Champagne J , et al. Radiofrequency ablation vs antiarrhythmic drugs as first‐line treatment of paroxysmal atrial fibrillation (RAAFT‐2): a randomized trial. JAMA. 2014;311(7):692–700. 10.1001/jama.2014.467 24549549

[2] Mont L , Bisbal F , Hernandez‐Madrid A , Perez‐Castellano N , Vinolas X , Arenal A , et al. Catheter ablation vs. antiarrhythmic drug treatment of persistent atrial fibrillation: a multicentre, randomized, controlled trial (SARA study). Eur Heart J. 2014;35(8):501–507. 24135832 10.1093/eurheartj/eht457 PMC3930872

[3] Nademanee K , McKenzie J , Kosar E , Schwab M , Sunsaneewitayakul B , Vasavakul T , et al. A new approach for catheter ablation of atrial fibrillation: mapping of the electrophysiologic substrate. J Am Coll Cardiol. 2004;43(11):2044–2053. 15172410 10.1016/j.jacc.2003.12.054

[4] Willems S , Klemm H , Rostock T , Brandstrup B , Ventura R , Steven D , et al. Substrate modification combined with pulmonary vein isolation improves outcome of catheter ablation in patients with persistent atrial fibrillation: a prospective randomized comparison. Eur Heart J. 2006;27(23):2871–2878. 16782716 10.1093/eurheartj/ehl093

[5] Verma A , Jiang CY , Betts TR , Chen J , Deisenhofer I , Mantovan R , et al. Approaches to catheter ablation for persistent atrial fibrillation. N Engl J Med. 2015;372(19):1812–1822. 25946280 10.1056/NEJMoa1408288

[6] Inoue K , Hikoso S , Masuda M , Furukawa Y , Hirata A , Egami Y , et al. Pulmonary vein isolation alone vs. more extensive ablation with defragmentation and linear ablation of persistent atrial fibrillation: the EARNEST‐PVI trial. Europace. 2021;23(4):565–574. 33200213 10.1093/europace/euaa293

[7] Kistler PM , Chieng D , Sugumar H , Ling LH , Segan L , Azzopardi S , et al. Effect of catheter ablation using pulmonary vein isolation with vs without posterior left Atrial Wall isolation on atrial arrhythmia recurrence in patients with persistent atrial fibrillation: the CAPLA randomized clinical trial. JAMA. 2023;329(2):127–135. 36625809 10.1001/jama.2022.23722 PMC9856612

[8] Masuda M , Inoue K , Tanaka N , Watanabe T , Makino N , Egami Y , et al. Long‐term impact of additional ablation after pulmonary vein isolation: results from EARNEST‐PVI trial. J Am Heart Assoc. 2023;12(17):e029651. 37642022 10.1161/JAHA.123.029651 PMC10547359

[9] Duytschaever M , Demolder A , Phlips T , Sarkozy A , El Haddad M , Taghji P , et al. PulmOnary vein isolation with vs. without continued antiarrhythmic drug treatment in subjects with recurrent atrial fibrillation (POWDER AF): results from a multicentre randomized trial. Eur Heart J. 2018;39(16):1429–1437. 29211857 10.1093/eurheartj/ehx666

[10] Fujiki A , Tsuneda T , Sakabe M , Nakagawa K , Mizumaki K , Hirai T , et al. Maintenance of sinus rhythm and recovery of atrial mechanical function after cardioversion with bepridil or in combination with aprindine in long‐lasting persistent atrial fibrillation. Circ J. 2004;68(9):834–839. 15329504 10.1253/circj.68.834

[11] Okumura Y , Watanabe I , Iso K , Nagashima K , Sonoda K , Sasaki N , et al. Clinical utility of automated ablation lesion tagging based on catheter stability information (VisiTag module of the CARTO 3 system) with contact force‐time integral during pulmonary vein isolation for atrial fibrillation. J Interv Card Electrophysiol. 2016;47(2):245–252. 27278517 10.1007/s10840-016-0156-z

[12] Wakamatsu Y , Nagashima K , Watanabe I , Watanabe R , Arai M , Otsuka N , et al. The modified ablation index: a novel determinant of acute pulmonary vein reconnections after pulmonary vein isolation. J Interv Card Electrophysiol. 2019;55(3):277–285. 30607666 10.1007/s10840-018-0501-5

[13] Nakahara S , Hori Y , Kobayashi S , Sakai Y , Taguchi I , Takayanagi K , et al. Epicardial adipose tissue‐based defragmentation approach to persistent atrial fibrillation: its impact on complex fractionated electrograms and ablation outcome. Heart Rhythm. 2014;11(8):1343–1351. 24793457 10.1016/j.hrthm.2014.04.040

[14] Takahashi K , Okumura Y , Watanabe I , Nagashima K , Sonoda K , Sasaki N , et al. Anatomical proximity between ganglionated plexi and epicardial adipose tissue in the left atrium: implication for 3D reconstructed epicardial adipose tissue‐based ablation. J Interv Card Electrophysiol. 2016;47(2):203–212. 27072363 10.1007/s10840-016-0130-9

[15] Park JW , Yu HT , Kim TH , Uhm JS , Joung B , Lee MH , et al. Mechanisms of long‐term recurrence 3 years after catheter ablation of atrial fibrillation. JACC Clin Electrophysiol. 2020;6(8):999–1007. 32819537 10.1016/j.jacep.2020.04.035

[16] Fink T , Schlüter M , Heeger CH , Lemes C , Maurer T , Reissmann B , et al. Stand‐alone pulmonary vein isolation versus pulmonary vein isolation with additional substrate modification as index ablation procedures in patients with persistent and long‐standing persistent atrial fibrillation: the randomized Alster‐lost‐AF trial (ablation at St. Georg Hospital for Long‐Standing Persistent Atrial Fibrillation). Circ Arrhythm Electrophysiol. 2017;10(7):e005114. 10.1161/CIRCEP.117.005114 28687670

[17] Yue L , Feng J , Gaspo R , Li GR , Wang Z , Nattel S . Ionic remodeling underlying action potential changes in a canine model of atrial fibrillation. Circ Res. 1997;81(4):512–525. 9314832 10.1161/01.res.81.4.512

[18] Nishida K , Fujiki A , Sakamoto T , Iwamoto J , Mizumaki K , Hashimoto N , et al. Bepridil reverses atrial electrical remodeling and L‐type calcium channel downregulation in a canine model of persistent atrial tachycardia. J Cardiovasc Electrophysiol. 2007;18(7):765–772. 17472715 10.1111/j.1540-8167.2007.00833.x

[19] Yamashita T , Ogawa S , Sato T , Aizawa Y , Atarashi H , Fujiki A , et al. Dose‐response effects of bepridil in patients with persistent atrial fibrillation monitored with transtelephonic electrocardiograms: a multicenter, randomized, placebo‐controlled, double‐blind study (J‐BAF study). Circ J. 2009;73(6):1020–1027. 10.1253/circj.cj-08-1061 19359813

[20] Kondo T , Miake J , Kato M , Ogura K , Iitsuka K , Yamamoto K . Impact of postprocedural antiarrhythmic drug therapy with bepridil on maintaining sinus rhythm after catheter ablation for persistent atrial fibrillation. J Cardiol. 2016;68(3):229–35. 26654806 10.1016/j.jjcc.2015.09.012

[21] Yakabe D , Fukuyama Y , Araki M , Nakamura T . Responsiveness to bepridil predicts atrial substrate in patients with persistent atrial fibrillation. J Arrhythm. 2021;37(1):79–87. 33664889 10.1002/joa3.12492 PMC7896463

[22] Kornej J , Hindricks G , Kosiuk J , Arya A , Sommer P , Husser D , et al. Renal dysfunction, stroke risk scores (CHADS2, CHA2DS2‐VASc, and R2CHADS2), and the risk of thromboembolic events after catheter ablation of atrial fibrillation: the Leipzig heart center AF ablation registry. Circ Arrhythm Electrophysiol. 2013;6(5):868–874. 24047706 10.1161/CIRCEP.113.000869

[23] Usuda K , Kato T , Tsuda T , Tada H , Niwa S , Usui S , et al. Impact of sinus rhythm maintenance on major adverse cardiac and cerebrovascular events after catheter ablation of atrial fibrillation: insights from AF frontier ablation registry. Heart Vessel. 2022;37(2):327–336. 10.1007/s00380-021-01929-5 34524497

[24] Shiga T , Suzuki A , Naganuma M , Hosaka F , Shoda M , Hagiwara N . Clinical outcome in patients with paroxysmal or persistent atrial fibrillation receiving bepridil. Circ J. 2011;75(6):1334–1342. 21483159 10.1253/circj.cj-10-1084

